# Trends and Patterns of Cancer Mortality in North China (Hebei Province), 1973–2013

**DOI:** 10.1038/s41598-017-18715-x

**Published:** 2018-01-10

**Authors:** Di Liang, Daojuan Li, Jianghui Liu, Jing Jin, Jin Shi, Baoen Shan, Yutong He

**Affiliations:** Cancer Institute in Hebei Province, the Fourth Hospital of Hebei Medical University, Shijiazhuang, 050011 Hebei Province China

## Abstract

Little was known about the cancer burden for the last 40 years in middle-income province in China. This study aimed to assess the overall, cause-specific mortality cancer trend and disability-adjusted life years (DALYs) between 1973–1975 and 2011–2013 in North China (Hebei Province). The collected data were stratified by 5-year age groups, gender and different types of cancer. We found that mortality from cancer showed an upward trend in the 1973–2013. The mortality rate of 0–79 year-old in 2011–2013 was lower than that in other periods. It was about two times higher for the 80+ age group than it was in 1973–1975. The cancer pattern in 4 periods presented differently. Esophagus cancer ranked the first in 1973–1975, whereas in 2011–2013, the most common cancer was lung cancer. DALYs also showed an increasing cancer burden in Hebei Province. This study is the first to analyze cancer burden for the last 40 years in a middle-income province. It could provide a baseline for assessment of effectiveness of cancer prevention and control. Esophagus cancer had a significant declining trend because of endoscopy screening program. Enhancing screening programs in those aged 40–69 year-old is necessary for reducing the cancer burden.

## Introduction

Cancer is the second leading cause of death globally, followed by cardiovascular diseases^1^. From 2005 to 2015, the age-standardized death rates for all kinds of cancer decreased in most developed countries, but the cancer burden from the main types of cancer was still heavy^2^. In 2015, approximately 16% of all deaths (8.7 million) worldwide were due to cancer^2^. And in China, cancer had been the most leading cause of death since 2010^3^. The *China Statistical Yearbook in 2016* indicated that cancer was the most common cause of death in rural and urban areas, accounting for 26.44% and 23.22% of all deaths, respectively^4^. In Hebei Province, a middle-income province, cancer also places a large burden on the health system. As the cause of death, cancer contributed to nearly 22% of all total deaths^5^. An increasing life expectancy, the growth in the aging population and high economic and social burdens contribute to increasing the incidence and mortality rate for most types of cancer.

Since the 1990s, the disability-adjusted life year (DALY) has been a common major metric for measuring the burden of disease worldwide. The sum of DALYs across the population, or the burden of disease, can be thought of as a measurement of the gap between the current health status and an ideal health situation in which the entire population lives to an advanced age, free of disease and disability^6,7^. It is the sum of the years of life lost due to premature mortality (YLL) and the years lost due to disability (YLD). DALYs have become a leading indicator of global burden of disease (GBD) research^6^. Not only is this metric conducive to finding public health problems, but it also facilitates the comparison of different regional burdens of one disease^1,8^. We would like to combine two metrics, the DALY and the mortality rate, to accurately estimate the burden of cancer in Hebei Province.

With a relatively developing economy, Hebei Province is located in North China (north latitude: 36°05′–42°37′, east longitude: 113°11′–119°45′) along the Taihang Mountain Chain. Additionally, it represents approximately 6% of the national population. The cancer registry in Hebei Province was established in 2009. Now, it covers 31 population-based cancer registries, with approximately 16.15% (11,184,118 households) of the total population. Furthermore, Hebei Province has participated three times in the national survey of all causes of death, conducted in 1973–1975, 1990–1992 and 2004–2005^9–12^.

In this study, the authors attempt to identify an up-to-date picture of the trends and patterns in cancer mortality for the last 40 years. Additionally, it is the first study to characterize the DALYs of cancer in different periods. This study can also assess the effect of cancer control and prevention in our province. It can be used by clinical, public health professionals and policy makers to inform targeted strategies to decrease the cancer burden and to improve health.

## Results

### Trend of cancer mortality rate between the 1970s and the 2010s

**Figure 1:**
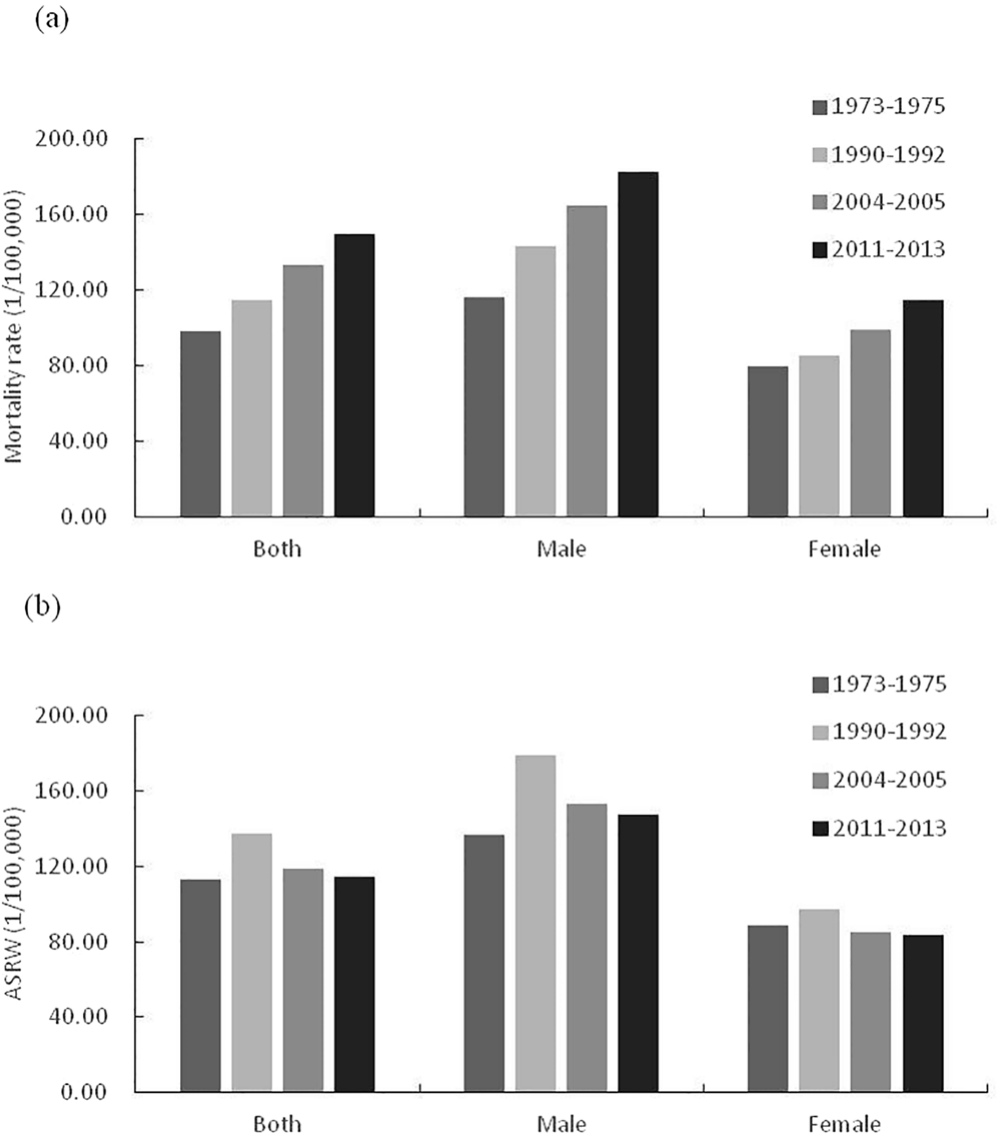
Mortality rate (**a**) and age-standardized mortality rate (**b**) in 1973–2013.

In 1973–1975, the age-standardized mortality rate was 112.73 per 100,000 population, whereas it was 114.42 per 100,000 population in 2011–2013. From 1990 to 2013, the adjusted mortality showed a slightly decreasing trend, but the adjusted mortality in 2011–2013 was also higher than that it in 1973–1975. The crude mortality rate increased by 51.57% between 1973 and 2013. It rose from 98.52 deaths per 100,000 population in 1973–1975 to 149.33 deaths per 100,000 population in 2011–2013. (Table 1 and Fig. 1).

**Table 1:** Mortality of cancer in Hebei province, 1973–2013.

	1973–1975			1990–1992			2004–2005			2011–2013		
	Both	Male	Female	Both	Male	Female	Both	Male	Female	Both	Male	Female
Estimate Deaths	141049	85173	55876	214015	136136	77879	180996	114580	66416	326465	203530	1122935
Crude rates (1/10^5^)	98.52	116.43	79.80	114.90	143.15	85.17	132.91	164.91	99.07	149.33	182.62	114.89
ASRC (1/10^5^)	112.65	136.21	89.32	137.58	179.4	97.85	119.3	153.89	85.74	114.93	147.78	84.45
ASRW (1/10^5^)	112.73	136.68	88.95	137.14	179.22	97.01	118.89	153.54	85.07	114.42	147.72	83.67

ASRC: Age-standardized rate by Chinese standard population (2000);.

ASRW: Age-standardized rate based on the world standard population.

### Change of median age for deaths from cancer

**Figure 2:**
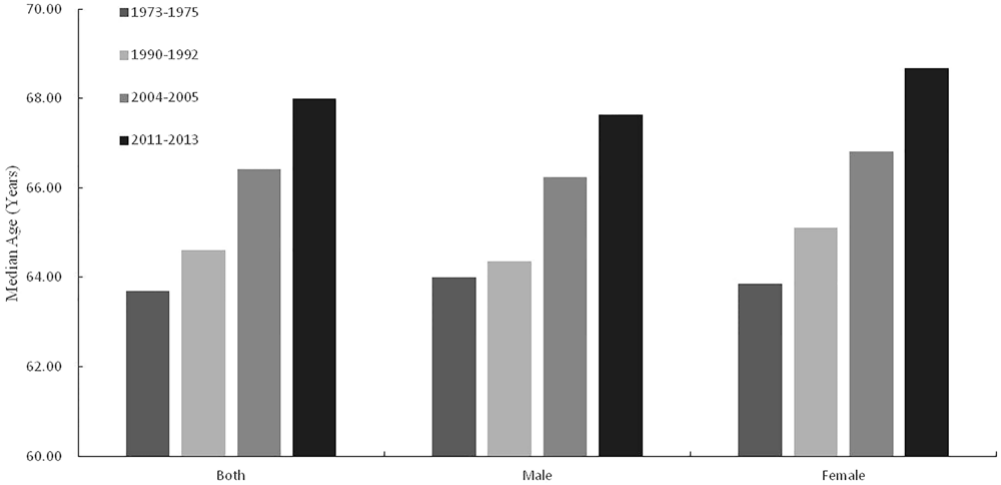
Median age of cancer deaths by sex during the period from 1973 to 2013.

The median age for cancer deaths increased from 1973–1975 to 2011–2013. In 1973–1975, the total median age of cancer deaths (25^th^ percentiles, 75^th^ percentiles) was 63.70 (59.58, 71.65) years old. It was 64.61 (55.94, 70.97) in 1990–1992, 66.42 (55.97, 74.11) in 2004–2005 and 67.99 (59.93, 76.89) in 2011–2013. The total median age increased 4.29 years. For males, the median age of cancer deaths rose 3.63 years, from 64.00 (55.15, 71.33) years in 1973–1975 to 67.63 (58.35, 76.40) in 2011–2013. For females, it rose 4.81 years, from 63.86 (58.10, 72.16) in 1973–1975 to 68.67 (57.86, 77.66) in 2011–2013 (Fig. 2).

### Age-specific mortality rate in four periods

**Figure 3:**
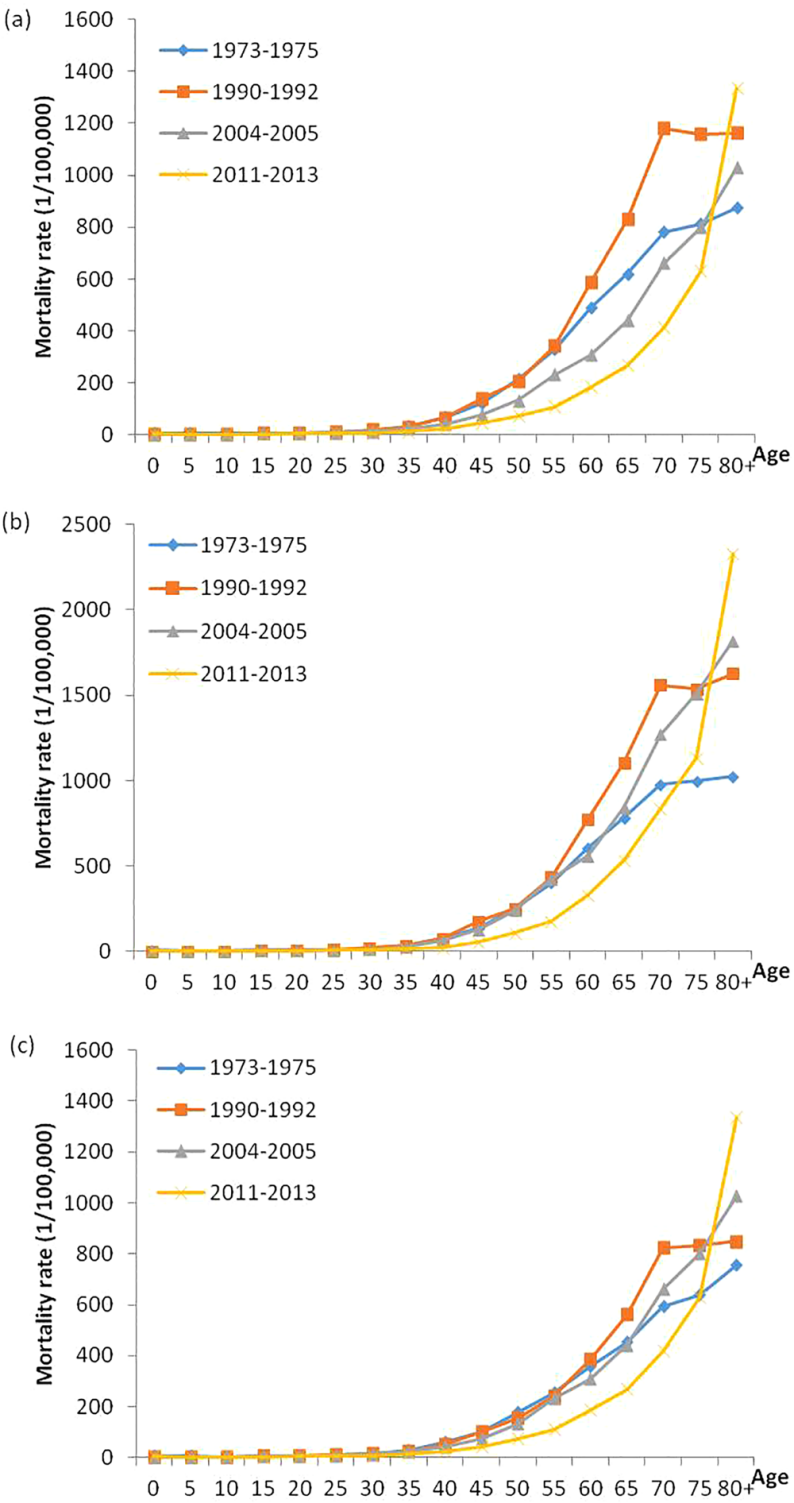
Change in age-specific mortality rate from cancer in both sexes (**a**), males (**b**) and females (**c**) during the period from 1973 to 2013.

In four periods, the age-specific mortality rate was low before the age of 40 and dramatically increased up to the 80+ age group. The age group of those over 80 years old reached the peak, except in 1990–1992, but the mortality rate of the 80+ age group maintained the highest rate in 1990–1992. Figure 3 showed that the last period had a lower mortality rate before the 75 age group. However, the rate for the age group of those over 80 years old in 2011–2013 was approximately two times higher than that in 1973–1975. Males and females had a similar trend in the age-specific mortality rate (Fig. 3).

### Change of the cause-specific mortality rate during the last 40 years

**Figure 4:**
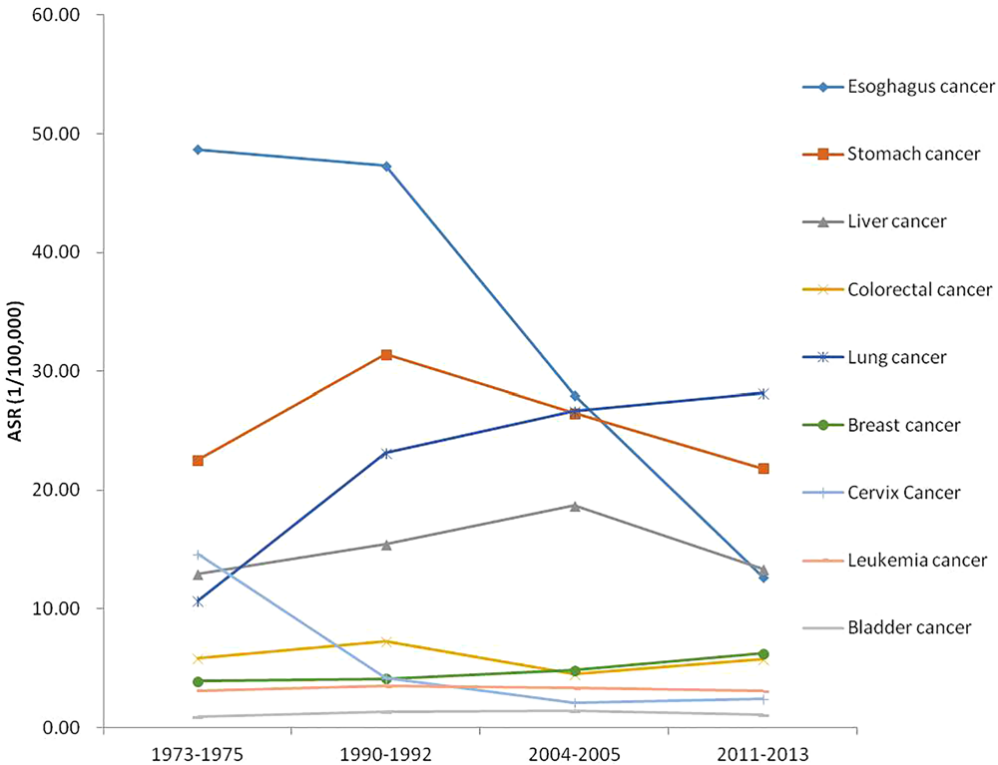
Change of cause-specific mortality rate in 1973–2013.

The findings showed differences in the mortality rates of the main types of cancer from 1973 to 2013. Figure 4 showed that several types of cancer had demonstrated a downward trend, including esophagus cancer, stomach cancer and cervix cancer. The age-standardized esophagus cancer mortality rate dramatically fell from 48.69/100,000 in the 1970s to 12.70/100,000 in 2011–2013, decreasing by 73.94%. Stomach cancer decreased from the 1990s to 2011–2013, changing -30.47%. Mortality from cervix cancer declined 83.50% from 1973 to 2013. However, an increasing trend for lung cancer and breast cancer was observed during four periods. For lung cancer, the age-standardized mortality rate was 10.69/100,000, 23.17/100,000, 26.64/100,000 and 28.15/100,000 in 1973–1975, 1990–1992, 2004–2005 and 2011–2013, respectively, increasing 163.33%. Breast cancer rose from 3.89 deaths per 100,000 population in the 1970s to 6.24 deaths per 100,000 population (Fig. 4).

### Cancer burden (DALY) from 1973 to 2013

The burden of cancer in 1973–1975 was 17.8 DALYs per 1,000 population. The YLL rate was 16.6/1,000, accounting for 92.2% of the total DALYs, and the YLD rate was 1.2/1,000, accounting for 7.8%. Over time, cancer caused 17.8, 20.2 and 21.3 DALYs per 1,000 population in 1990–1992, 2004–2005 and 2011–2013, respectively, of which 94.8%, 91.3%, 92.0% came from YLLs and 5.2%, 8.7%, 8.0% from YLDs. By 2011–2013, the cancer burden had increased 19.66% compared to the 1970s. In every period, cancer in males contributed a higher burden than cancer in females. The DALYs in male produced 18.0, 21.5, 24.6 and 24.3 per 1,000 in 1973–1975, 1990–1992, 2004–2005 and 2011–2013, respectively. Additionally, the DALYs in females were 14.3, 14.0, 15.6 and 18.3 per 1,000, respectively (Table 2).

**Table 2:** DALYs per 1,000 persons for cancer in Hebei province.

Year	Sex	YLLs	YLL(%)	YLDs	YLD(%)	DALYs
1973–1975	Both	16.6	92.2	1.2	7.8	17.8
	Male	17.0	94.5	1.0	5.5	18.0
	Female	12.8	89.1	1.6	10.9	14.3
1990–1992	Both	16.9	94.8	0.9	5.2	17.8
	Male	20.4	95.2	1.1	4.8	21.5
	Female	13.2	94.3	0.8	5.7	14.0
2004–2005	Both	18.5	91.3	1.8	8.7	20.2
	Male	22.2	90.5	2.3	9.5	24.6
	Female	14.5	92.8	1.1	7.2	15.6
2011–2013	Both	19.6	92.0	1.7	8.0	21.3
	Male	23.2	95.9	1.0	4.1	24.2
	Female	15.9	86.9	2.4	13.1	18.3

YLL: year of life lost due to premature mortality;.

YLD: year lost due to disability;.

DALY: disability-adjusted life year.

**Figure 5:**
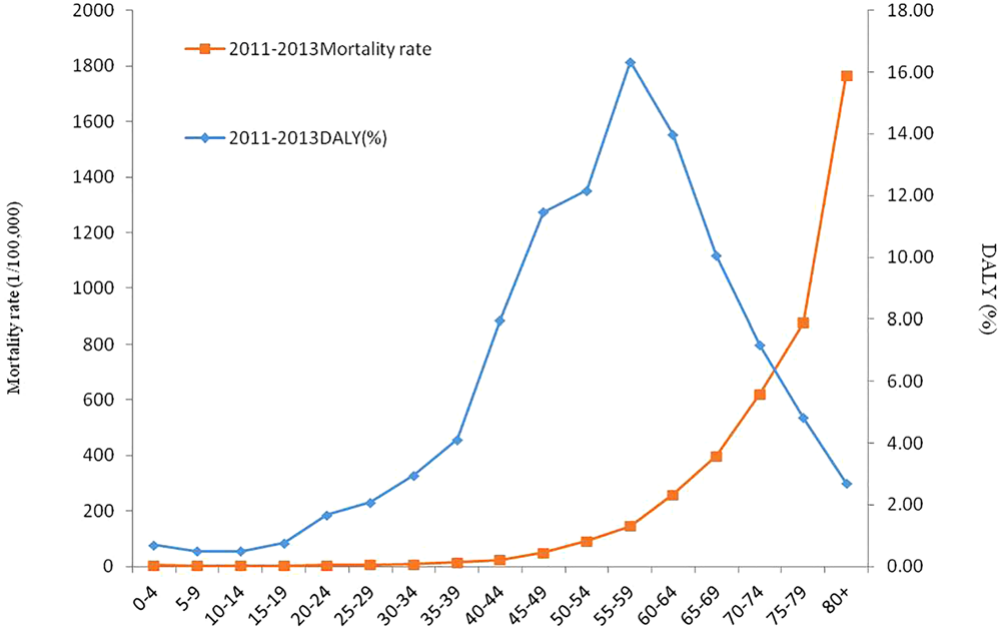
Age-specific DALY percentage and age-specific mortality rate in 2011–2013.

From the age-specific DALYs in 2011–2013, the health loss increased from the 35–39 age group and peaked at the 55–59 age group, accounting for 16.35% of total DALYs. From 45 years old to 69 years old, the percentage of DALYs was 64.03% of all DALYs (Fig. 5).

## Discussion

Using the three times survey of all causes of death and the data in cancer registries, this study was the first systematic statistical analysis assessing the overall cancer burden for the last 40 years in North China (Hebei Province). Identifying the cancer mortality, the cause-specific mortality rate and the change in DALYs in recent decades can makes it possible to understand the worsening burden, the effectiveness of screening programs and inadequate access to quality treatment. Although previous estimates of the incidence and mortality of cancer in North China have been reported, they were based on data from a specific year^13,14^. From 1973 to 2013, the age-standardized mortality rate increased. However, crude cancer mortality increased from 98.52/100,000 to 149.33/100,000, rising 51.57%. Additionally, DALYs increased 19.66% during the last 40 years. Thus, cancer is also a priority public health challenge in middle-income provinces (Hebei Province) in North China.

This study had found that the age-standardized mortality rate slightly decreased from 1990–1992, but the adjusted rate in 2011–2013 (114.42 per 100,000 population) was also higher than that in 1973–1975 (112.73 per 100,000 population). In the period from 1973 to 2013, the estimated cancer death cases in each year of the four periods were 47,877, 71,444, 90,484 and 108,821 cases. Adjusted mortality from cancer in Hebei Province in the 2010s was higher than the global level (102.4/100,000)^15^. It was also higher than the national level in 2013 (108.94/100,000)^16^. The YLLs in the 2010s were 1.40 times higher than those in 1973–1975. Additionally, YLLs showed an upward trend regardless of gender. Thus, we have faced a heavy challenge due to the ascending burden of cancer in our province. According to report by the National Cancer Center, the mortality rate in China demonstrated an ascending trend during the period from 1973–1975 (75.60/100,000) to 2013^9,16^. The cancer burden trend in Hebei Province was similar to the national trend and the trend of other provinces^12,16,17^. But it was not consistent with that of developed countries, such as the United States and Europe^18–20^. The cause of the increased cancer burden had two factors. On one hand was the aging population. According to the annual statistical report for Hebei Province, the life expectancy increased 4.0 years (2.9 years for males and 5.6 years for females) from 1990 to 2013^21,22^. From the 1970s to the 2010s, the demographic structure had experienced a shift from an adult type to an elderly type. In the 1970s, the population of those 60 years old and older accounted for 9.49% of the total population. The percentage of those aged 80+ in the aging population (≥60 years old) was 7.07%. In contrast, in the 2010s, the aging population proportion reached 16.3%, which was higher than the national level. It was 1.71 times higher than that in the 1970s. The percentage of the population that was more than 80 years old was 12.4% in the aging population^21,23^, which also presented an upward trend. The percentage of the aging population continues to expand, and the increasing speed of those aged 80+ years old is faster than the aging population. The median age of cancer deaths grew 4.29 years. For males, it increased 3.63 years and for females, 4.81 years. Another factor concerned unhealthy lifestyle and the diet structure, increasing obesity, air pollution and other factors that could enhance the cancer burden^24,25^. For example, smoking was associated with an increased risk of esophagus cancer (Risk ratio = 1.67) and lung cancer^26–28^. There were approximately 250 million smokers in China, rising to 367 million in 2012. In Hebei Province, the tobacco consumption rate was 48.09% in males, and the prevalence has been increasing in recent decades. The drinking rate was 41.1% in both sexes and was 71.8% in males, higher than that in the 1980s^21^. Therefore, these reasons could be responsible for the increase in the cancer burden in Hebei Province.

We found that the pattern of cancer mortality changes varied differently by type in recent decades. Some infection-related cancers (for example, esophagus cancer, stomach cancer and cervix cancer) declined. Whereas some cancers linked to a westernized lifestyle and kinds of pollution, such as breast cancer and lung cancer, displayed an upward tendency. In 1973–1975, esophagus cancer, stomach cancer and liver cancer were ranked as the top three among all types of cancer. In contrast, in 2011–2013, lung cancer, stomach cancer and liver cancer were the major causes of cancer-related death. Comparing 1973–1975 and 2011–2013, the adjusted mortality rate of lung cancer and breast cancer showed a 163.33% and 60.41% increase, respectively. Additionally, esophagus cancer and cervix cancer dropped more quickly than other types of cancer, with a 73.94% and 83.37% decrease, respectively.

In 1973–1975, in China, the adjusted mortality rate from esophagus cancer was 23.20/100,000, but in Hebei Province, it was 2.10 times higher (48.69/100,000). Thus, Hebei Province was recognized as a high-risk area of esophagus cancer from the first national survey of all causes of death. There was a deep relationship between esophagus cancer and geographic factors, unhealthy lifestyles, alcohol consumption, poor oral health and pickled food consumption^29–32^. Through efforts in esophagus cancer prevention and control over the last 40 years, mortality from esophagus cancer had a dramatically downward trend in our province. The change in esophagus cancer (a 73.94% decrease) was faster than the national level (a 41.64% decrease) over the last 40 years. Endoscopy technology in those aged 40–69 and enhancing primary prevention have played an important role in decreasing the mortality rate. Since 2000, a national screening program using endoscopy with mucosal iodine staining and index biopsy combined with pathological examination for confirming and staging the disease has become available in 17 areas in Hebei Province. Additionally, the early detection rate was nearly 80%, and the checkout rate from cancer was approximately 2%. The study by Wei *et al*. shows that the sensitivity and specificity of endoscopy are 96% and 63%, respectively^33^. It has been one of the effective method for reducing the mortality and incidence of esophagus cancer. Targeted prevention and local health care systems have also ensured that advances in cancer control have been accessible in Hebei province. It has been estimated that approximately 60% of cancer deaths would be decreased by reducing exposure to risk factors^25^. First, enhancing the hygiene system has been helpful in reducing mortality from esophagus cancer. In the 1970s, high-risk areas of esophagus cancer incidence and mortality (such as Cixian and Shexian) used rain water for drinking. The levels of nitrate, nitrite, nitrogen, and ammonia in drinking water of high-risk areas significantly exceeded the national standards for drinking water^34^. Now, the majority of residents had groundwater for drinking. Second, efforts to enhance the awareness of cancer prevention were effect for cancer control, such as the decreasing consumption of pickled vegetables, the intake of more fruits and vegetables and traditional dietary habits^35–37^. These efforts had been able to contribute to reducing the burden of esophagus cancer. In the 2010s, the mortality rate (12.70/100,000) was also higher than the worldwide level (5.9/100,000) and the national level (9.98/100,000)^15,16^. Thus, in the future, endoscopy and primary prevention methods and decreasing risk factors will be more fully utilized for the prevention and control of esophagus cancer in China and worldwide.

Facilitating secondary prevention (earlier detection, earlier diagnosis and earlier treatment) may hold the greatest potential for having a more immediate impact on the existing burden of cancer and extending the 5-year survival quality and rate, particularly for lung cancer and breast cancer. Lung cancer and breast cancer presented a faster growth trend of mortality, with a 163.33% and 60.41% increase, respectively. A large randomized controlled trial, the national Lung Cancer Screening Trial, had indicated that low-dose computed tomography (LDCT) screening for lung cancer could reduce mortality by 20%^38^. In developed Asian countries such as Hong Kong in China and Singapore, where widespread mammography screening and improved access to recent advances in treatment had been available, more than 70% of breast cancers were diagnosed at stage I and II. Additionally, the overall breast cancer survival rates exceed 85%^39–42^. In Hebei Province, the cancer burden in these age groups for those aged 40–69 accounted for more than 70% of all DALYs. Those aged 55–59 possessed the most disability-adjusted lost years. Because, there were many years between actual age (40–69 years old) and life expectancy in Hebei Province. And more incidence and mortality cases occurred in these age groups. So patients in aged 40–69 had a heavier cancer burden than for the other groups. Thus, we should more focus on a target population of those aged 45–69 to perform LDCT and mammography screening for lung cancer and breast cancer prevention. Doing so could obtain economic benefits, a high survival rate and quality of life and a reduced cancer burden. It was also similar to other cancer screening programs in developed countries^38,39^.

This study had some strengths and limitations. Using a population-based database for the last 40 years, it could provide an up-to-date cancer burden for a middle-income province in China. Analyzing the change in the cancer mortality trend, it could assess the effect of cancer prevention and early detection screening in Hebei Province, such as endoscopy with mucosal iodine staining. The effective screening method and prevention could extend to all areas in China and even worldwide. The limitations are that the study used data from four periods for analysis, and the quality and quantity of the data in recent years were better than those in 1973–1975 and 1990–1992. There may be a minor problem in terms of comparability and representativeness.

## Materials and Methods

### Data source

This study used the identified death records from three time points at which the survey of all causes of death was conducted and the database of the Hebei Province cancer registry. The first time survey of all death causes, hold in 1973–1975, covered all population in Hebei Province. The second and the third time death cause’s survey in 1990–1992 and 2004–2005 were population-based studies and used stratified sampling method. Cancer registry’s data in 2011–2013 was also stratified sampling by different sociodemorgraphic areas. The data covered death reports that occurred from 1973–1975, with information by age, sex and sociodemorgraphic being provided by the cancer registry. The four times’ data could represent Hebei Province’s condition in each period. The reported cases were from multiple sources, including local hospitals, community health centers, and the Urban Resident Basic Medical Insurance Program and the New Rural Cooperative Medical Scheme.

In 1973–1975, a nationwide retrospective survey on the causes of mortality was conducted in 29 provinces. At that time, all 153 cities and counties in Hebei Province, covering 143.17 million (143,174,856) people, were included^9^.

The second national retrospective stratified sampling survey on all causes of death was conducted in 1990–1992. A total of 21 cities and counties, covering 19.41 million (19,414,821) people from Hebei Province, were enrolled as sampling areas, representing approximately 10.41% of the total population in Hebei Province^10,11^.

The third national retrospective stratified sampling survey on all causes of death, performed from 2004–2005, was conducted in 31 provinces/municipalities/autonomous regions and included Hebei Province. A total of 18 cities and counties were selected as sampling areas, covering 13.79 million people and 10.10% of the total population of Hebei Province^12^.

We collected the death records of 21 cancer registries from 2011 to 2013. All data were selected after quality control according to the data quality criteria of the National Cancer Center and the all-cause death monitor system. In the 21 cancer registries, there were 20 all-cause death monitor registries, and they were able to have regional representation in Hebei Province. The database for 2011–2013 covered 20,744,210 households, representing approximately 9.48% of the total population in Hebei Province^5,13,14^.

### Data quality control

To ensure the high quality of the data across the four time points of the survey, quality control was performed in the following aspects: organization and leadership, investigation and design, investigators, on-site investigation, data collation, the data entry stage and processing data, and data analysis, among others. According to the data of the cancer registries and the all-cause death monitor program handbook, unified training and examination for the investigation team was performed. Additionally, after the investigation, two percent of the data were checked, such as the names, sex, age at death, disease diagnosis, grade of the disease and other key variables. Then, we conducted the household survey to check information, and the qualified rate reached 98% or more. Two percent of the death records were redefined as the primary cause of death, and five percent of eth death records were recoded. The coincidence rate of the accurate primary cause of death and the coding accuracy reached more than 99%. Data in accordance with the above quality control criteria were included in the final statistical analysis.

### Mortality rate calculating

The population of Hebei Province was stratified by sex (male/female) and age groups (0–4, 5–79 by 5 years old, 80+ years old). The world age-standardized rate (ASRW) and Chinese age-standardized rate (ASRC) were calculated by Segi’s population and the Chinese population in 2000.

Age-standardized rate are calculated according to the following formulas:

1 $${\rm{Adjusted}}\,{\rm{rate}}=\frac{{\boldsymbol{\Sigma }}\,{\rm{Standard}}\,{\rm{population}}\,{\rm{in}}\,{\rm{corresponding}}\,{\rm{age}}\,{\rm{group}}\ast {\rm{age}}-{\rm{specific}}\,{\rm{rate}}}{{\boldsymbol{\Sigma }}\,{\rm{Standard}}\,{\rm{population}}}\ast 100,000$$

### DALYs calculating

DALYs for a specific cause of death are the sums of the years of life lost due to cancer death and the years lost due to disability^35^. The following formula was used for the calculation of the DALYs: DALYs = YLLs + YLDs. Calculating the YLLs and YLDs was performed using the following formula:

2 $${\int }_{\alpha }^{\alpha +L}Dcx{e}^{-\gamma (x-a)}{e}^{-\beta x}dx$$

It could be transformed to

3 $${\rm{N}}\frac{Dc{e}^{(-\beta \alpha )}}{{(\beta +\gamma )}^{2}}[{e}^{-(\beta +\gamma )(L+\alpha )}\,[-((\beta +\gamma )(L+\alpha )-1)-{e}^{(-\beta +\gamma )\alpha }[-1-(\beta +\gamma )\alpha ]]$$

where D is the disability weight^36^ and D is equal to 1 for cancer death, c is the age weighted adjustment factor (the GBD standard value is 0.1658), β is the parameter from the age-weighting function (the GBD standard value is 0.04), α is the age at onset or the age at death, γ is the discount rate (the GBD standard value is 0.03), and L is a standard loss function specifying the years of life lost for the age at onset or the age at death. The life expectancy data were from the 26^th^ level of the life table in the “Western” family model and research by WHO^43^.

### Statistical analysis

All data analyses were performed with SPSS (version 20, Inc, Chicago, IL, USA), SAS (version 9.2, SAS Institute Inc., Cary, NC, USA) and Ms-Excel (Excel, Microsoft Corp., Redmond, WA, USA). And we use DisMod II (Erasmus University, Netherlands) to access DALY value^44^.
